# PCOS and breast cancer risk: hormonal modulation and epidemiological trends

**DOI:** 10.1186/s12978-026-02355-4

**Published:** 2026-05-22

**Authors:** Jyolsna Ponnaratta Kunhiraman, Reyon Dcunha, Ganesh Venkatraman, Anujith Kumar, Satish Kumar Adiga, Nagarajan Kannan, Guruprasad Kalthur

**Affiliations:** 1 Division of Reproductive Biology, Department of Reproductive Science, Kasturba Medical College, Manipal Academy of Higher Education, Manipal, Karnataka 576104 India; 2 Sree Mookambika Centre for Research and Innovation, Sree Mookambika Institutes, Padanilam, Kulasekharam, Tamil Nadu 629161 India; 3 School of Biosciences and Technology, Vellore Institute of Technology, Vellore, 632014 Tamil Nadu India; 4 Manipal Institute of Regenerative Medicine, Manipal Academy of Higher Education, Manipal, Karnataka 576104 India; 5 Centre of Excellence in Clinical Embryology, Department of Reproductive Science, Kasturba Medical College, Manipal Academy of Higher Education, Manipal, Karnataka 576104 India; 6 Division of Experimental Pathology and Laboratory Medicine, Department of Laboratory Medicine and Pathology, Mayo Clinic, Rochester, MN 55905 USA; 7 Center for Regenerative Medicine, Mayo Clinic, Rochester, MN 55905 USA; 8 Mayo Clinic Cancer Center, Mayo Clinic, Rochester, MN 55905 USA

**Keywords:** Obesity, Infertility, Estrogen, Cancer, Controlled ovarian hyperstimulation

## Abstract

**Background:**

Hormonal signals intricately regulate breast growth and development. Any perturbations due to altered endocrine and metabolic conditions, such as in polycystic ovary syndrome (PCOS), may influence breast cancer risk. This review highlights the susceptibility of women with PCOS to breast cancer, with a focus on the potential contribution of its comorbidities, such as hyperandrogenism, obesity, insulin resistance, diabetes, menopausal status, inflammation, and use of ovulation induction agents to this association.

**Methods:**

This is a narrative review including articles and information collected from PubMed, Scopus, Google Scholar, and the World Health Organization (WHO) websites. Studies were selected based on the availability of an abstract and research publication in English. Reports related to PCOS, its characteristics (hyperandrogenism, hyperinsulinemia, insulin resistance, type 2 diabetes, obesity), fertility treatment, and their association with breast cancer were included.

**Results:**

The interrelation between PCOS and breast carcinoma remains ambiguous due to inconsistent and inconclusive epidemiological evidence. The comorbidities associated with the condition are reported to collectively or individually influence this association. Women with PCOS present anovulatory cycles and have a high necessity of undergoing ovulation induction, further altering hormonal dynamics. Evidence in the literature suggests that the number of treatment cycles, age at the onset of treatment, and cumulative doses of agents used for ovulation induction are a few other factors that could affect breast cancer risk, though the results are inconclusive.

**Conclusion:**

Existing literature suggests that PCOS and its characteristics may impact the process of breast development and possibly contribute to breast cancer, although the evidence is still conflicting. Moreover, temporary alterations of endocrine profile following fertility medications may have a significant influence on the breast cancer risk, although research has shown inconclusive evidence. Hence, robust experimental studies are required to understand the underlying mechanisms and the long-term health implications for women with PCOS due to the intricate association between these factors.

## Abstract (highlights)

Few studies suggest that an altered metabolic and endocrine profile in PCOS women predisposes them to breast cancer. However, current epidemiological evidence is inconclusive in establishing a strong link between PCOS and the risk of breast cancer. As PCOS women often depend on assisted reproductive technologies for infertility treatment, ovulation induction/ controlled ovarian stimulation protocols may further increase the health risks. The present article is a comprehensive overview highlighting the possible association with breast cancer risk in PCOS women undergoing assisted reproduction.

## Background

Polycystic ovary syndrome (PCOS) is the most common endocrinopathy in women of reproductive age, with a prevalence of 6—13% globally [1], among which up to 35% are reported from India [2, 3]. This metabolic condition is a major contributor to ovulatory dysfunction [4, 5] affecting approximately 25% of infertile couples [6, 7]. Women with PCOS exhibit several metabolic dysfunctions, such as obesity, hyperlipidaemia [8], insulin resistance [9] and hyperandrogenism [10]. The elevated androgen levels in these women disrupt the hypothalamus-pituitary-ovarian axis, resulting in increased luteinizing hormone (LH) levels, anovulation, and impaired ovarian function [4]. Consequently, women with PCOS display poor oocyte quality [11], compromised embryo development, endometrial abnormalities [12] and pregnancy complications [13], necessitating these patients to undergo ovulation induction or controlled ovarian hyperstimulation (COH) for assisted reproductive technology (ART).

Data from 194 countries reports that the global age-standardised incidence rate and disability-adjusted life years (DALY) for PCOS women, which is a comprehensive measure of premature mortality and disability, showed a slight increase of 1.45% and 1.91%, respectively [14]. The most notable increase in age-standardised PCOS incidence was found in regions with a middle socio-demographic index. However, 70% of women with PCOS remain undiagnosed, suggesting that the true incidence of the condition may be significantly underestimated [1]. The definition of PCOS has evolved, with several sets of diagnostic criteria proposed by different expert groups [15, 16], varying levels of provider knowledge, and the absence of a standardised consensus present significant challenges in the management of women with PCOS [17]. These factors contribute to inaccurate diagnosis, resulting in both under- and overdiagnosis of the condition.

Breast development in females is a hormonally regulated process that begins at the embryonic stage and continues throughout puberty, with progressive changes during pregnancy and lactation [18]. Both progesterone and estrogen play a coordinated role in the growth and development of the normal breast. During puberty, the initial increase in estrogen and progesterone levels triggers the proliferation of mammary cells, resulting in a noticeable enlargement of the breast volume. The initial rapid growth phase concludes on attaining sexual maturity, followed by transient surges during the menstrual cycle. The major proliferative phase, characterised by high circulating levels of progesterone and estrogen, corresponds to the mid-luteal phase of the menstrual cycle and marks the onset of epithelial alveolar bud formation. The increased level of mitotic activity exhibited by progesterone during this phase is correlated with mammary cell proliferation [19]. On the contrary, during the menstruation phase or late luteal phase, the mammary gland epithelium undergoes regression. The newly formed alveolar buds prepare the organ for the subsequent menstrual cycle by undergoing apoptosis and remodelling of the tissue to their basic structure [20, 21]. These hormones orchestrate a tremendous wave of proliferative and morphological changes throughout pregnancy to promote the establishment of functional milk-producing alveolar structures in preparation for lactation, marking the second major phase of development. Finally, menopause is marked by the end of ovarian hormone production, during which fatty tissue and stromal cells gradually replace the breast's epithelial cell compartment [22].

Earlier reports have shown that PCOS can affect the normal breast development process [23–27]. Women with PCOS often experience a chronic elevation in the estrogen-to-progesterone ratio, similar to the hormonal fluctuations observed during the menstrual cycle [23]. This imbalance increases the risk of fibrocystic breast changes [24], which may exacerbate in PCOS women due to hyperandrogenism, where elevated androgen levels inhibit progesterone and promote excessive mammary epithelial cell proliferation [24–27]. Due to the abnormal endocrine levels and associated alterations in metabolic function, the link between PCOS and cancer risk remains a highly debated topic. Several epidemiological studies show no consistent evidence between PCOS and breast cancer risk [25, 27–32]. This inconsistency likely reflects heterogeneity in risk profiles, influenced by variations in hormonal milieu, obesity status, and the severity of insulin resistance among affected individuals. Under these conditions, PCOS women seeking ART may have a higher probability of breast cancer risk due to repeated ovulation stimulation and cumulative hormonal exposure. The purpose of this review is to recapitulate the predisposition of women diagnosed with PCOS to breast cancer risk, with special emphasis on the possible role of ovulation-inducing agents on the mammary gland cells.

## Methods

A comprehensive literature search was performed using PubMed, Scopus, Google Scholar, and the World Health Organization (WHO) website. Studies were selected based on the availability of an abstract and publications in English, and relevance to cancer and reproductive health. Specific keywords used in the search included “infertility,” “PCOS,” “anovulation,” “hyperandrogenism,” “hyperinsulinemia,” “obesity,” “genetic mutation,” “menopause,” “breast cancer,” “ovulation induction,” “metformin,” “letrozole,” “clomiphene citrate,” “controlled ovarian hyperstimulation,” “cancer” and “fertility treatments.” Articles that examined the association between PCOS or its clinical features and breast cancer, as well as studies exploring the underlying mechanisms of this relationship, were included in this review.

### PCOS and breast cancer risk

Breast cancer remains a significant global health concern, particularly among women of reproductive age [33]. According to the WHO, in 2022, breast cancer was the most common cancer type in women, with 2.3 million new cases and 670,000 deaths reported globally [34]. The 2020 report from Global Cancer Observatory (GLOBOCAN) identified breast cancer as the leading cause of cancer cases and deaths in India, accounting for an estimated 26.6% of new cancer diagnoses and 13.7% of all cancer-related deaths among women [35].

PCOS is characterised by complex endocrine and metabolic alterations, resulting from a combination of genetic and environmental factors [36], which are associated with elevated androgens, infertility [7] and cancer risk (Table 1). Overexpression of genes [for example, denn domain containing protein 1 A (DENND1A)] has been demonstrated to significantly stimulate the androgen pathway, resulting in a PCOS-like phenotype [48]. Mendelian randomisation analysis using genetic variants revealed that higher total blood testosterone, bioavailable testosterone, and reduced sex hormone binding globulin (SHBG) increase the risk of PCOS [49]. Therefore, elevated levels of androgens and other paracrine regulators, along with increased anti-Müllerian hormone (AMH) levels in PCOS [50], tend to prevent the spontaneous development of antral follicles that can potentially ovulate [51].

**Table 1:** Various cancer risks (except for breast cancer) reported in PCOS women

Cancer	Risk	Reference
Endometrium	Increase	[32, 37, 38]
	No risk	[39]
Ovary	No risk	[31, 32, 40]
	Increase post menopause	[41]
	Increase pre menopause	[42]
	Increase in nulliparous women	[43]
	Increase	[44]
	Decrease	[45]
Cervix	No risk	[46]
Kidney	Increase	[42, 47]
Brain	Increase	[47]
Colon	Increase	[47]
Thyroid gland	Increase	[42]
Pancreas	Increase pre menopause	[42]
Skeletal and hematopoietic system	Increase	[42]

A central element of the relationship between PCOS and breast cancer risk lies in steroid hormone imbalance, as mammary cell regulation depends on the interplay between estrogenic stimulation and androgenic inhibition [52–55]. The ovarian granulosa cell layer is responsible for the aromatisation of androgens to estrogens under the influence of follicle-stimulating hormone (FSH) and may act on estrogen-sensitive cells of the mammary gland, whereas dehydroepiandrosterone (DHEA) acts through the androgen receptor (AR) to inhibit cell growth [52, 53]. However, when androgen excess is sustained, estrogen-driven signalling predominates and is further amplified by growth factors such as epidermal growth factor (EGF) [54]. Additional endocrine disturbances commonly seen in PCOS, including reduced levels of SHBG, elevate the bioavailability of free estrogens and androgens, thereby magnifying their mitogenic activity in mammary cells [55]. However, epidemiological studies have observed heterogeneous results after evaluating the risk of breast cancer among women with PCOS [31, 47, 56, 57] (Table 2).

**Table 2:** Epidemiological studies associated with PCOS and breast cancer in women

PCOS diagnostic criteria	Sample size	Age range (years)	Follow-up period	Country/ Ethnicity	Risk	Major findings	Reference
Meta-analysis							
Not specified	45,470 from 8 studies (3 case–control—0.87 (95% CI, 0.44 to 1.31); 5 cohort—1.18 (95% CI, 0.93 to 1.43)	24—69	NA	Taiwan Denmark Italy Iran USA UK	No risk	NA	[28]
Rotterdam criteria, NIH, histological assessment, ICD codes, self-reported or no record	1,186,242 from 13 studies (5 case control; 8 cohort studies)	< 50 > 50	NA	Taiwan Japan USA Denmark UK Australia Italy Greece Iran	9% elevated risk in European women. Significant association for those over 50 years of age. Cumulative results from studies diagnosing PCOS based on clinical criteria and specialist doctors revealed a stronger association	More frequently reported among European women than their American and Asian counterparts. Increased risk may be linked to age, PCOS criteria, diverse lifestyles, diets, and healthcare behaviors in these communities.	[29]
NIH 1990 Criteria; Rotterdam Criteria; AE-PCOS Society Criteria – Hyperandrogenism; Ovulatory dysfunction (oligo- or amenorrhea); PCOS morphology	100,000 (3 case control; 7 cohort study; 1 meta-analysis)	Not specified	NA	Denmark Taiwan US Australia Italy Greece UK Japan	No risk	Anovulation reduces luteal hormone exposure (E2 & P4), potentially lowering breast cancer risk.	[25]
NIH Rotterdam 2003 AES 2006	23,842	20—75	NA	Not specified	No risk	NA	[58]
Rotterdam, National Institute of Health, Androgen Excess Society, International Classification of Diseases, and also a self-reported questionnaire	12,955 PCOS and 118,481 control	18—98	NA	Taiwan US UK Iran Australia Italy Georgia	No risk	Anovulation reduces luteal hormone exposure (E2 & P4), potentially lowering breast cancer risk.	[59]
Rotterdam criteria, non-standard criteria, and self-reported	3 case control; 4 cohort study	15—98	NA	Sweden China UK US Australia Iran Italy Greece Japan	No risk	Considered the influence of age and menopausal status, study design, and confounding factors (such as BMI). Low risk may be attributed to inhibition of breast growth and neoplasia by androgen.	[30]
Self-reported Rotterdam	919 PCOS 72054 control (2 case control; 1 cohort study)	20–74	NA	Caucasian	No risk	-	[40]
Cohort studies							
NIH or ICD-9-CM Oligo-ovulation, hyperandrogenism (clinical or biochemical), and exclusion of other disorders that can result in menstrual irregularity and hyperandrogenism	3,566 PCOS 14,264 control	PCOS: 27.04 (22.66–32.10) Control: 27.05 (22.72–32.10)	7.15 years (Median)	Taiwan	No risk	Monte Carlo method indicates no additional risk. Comorbidities assessed—Age, hypertension, diabetes mellitus, dyslipidemia, coronary artery disease, congestive heart disease, cerebrovascular disease, chronic pulmonary disease, urbanization, and monthly income.	[31]
Blood tests for luteinizing hormone, follicle-stimulating hormone, and testosterone levels, and or ultrasonography for polycystic ovaries	8155 PCOS 32,620 control	PCOS—27.7 ± 7 Control – 27.5 ± 7.9	PCOS – 6.0 ± 4.0 Control – 6.3 ± 4.0	Taiwan	No risk	Comorbidities assessed – hypertension, diabetes mellitus, hyperlipidemia, stroke, coronary artery disease, congestive heart failure and chronic obstructive pulmonary disease.	[32]
Danish version of the International Classification of Diseases (ICD) ICD-8 = 256.90 during 1977–1993, and ICD-10 = E28.2 during 1994–2012	12,070	9–49	5.7 years (median)	Danish	No risk	Population exhibited non-significant Standardized Incidence Ratio (SIR), tendency towards protective effect among women aged < 50 years and increased risk in women ≥ 50 years,	[47]
ICD-8 ICD-10 Rotterdam criteria	15,131 PCOS	28.1 (IQR: 24.4–32.3)	26 years (Median)	Danish	Increase	Co-variants assessed- education status, obesity, diabetes mellitus type II, use of hormonal contraceptives, infertility treatment (IVF), parity status. PCOS associated with increased risk of overall, ductal, and lobular breast cancer, restricted to postmenopausal women; no significant association in premenopausal women, possibly due to longer lifetime estrogen exposure from delayed menopause. Similar PCOS association with ductal and lobular cancer, consistent with comparable ER/PR expression.	[60]
Swedish register data	14,764	15—50 years	NA	Swedish	No risk	Covariates assessed including the highest achieved educational level, parity, parental cancer history, use of medications (metformin, oral contraceptives, and hormone replacement therapy), and diabetes	[42]
Definite PCOS (62%), histological evidence of PCO with clinical evidence of ovarian dysfunction; or (ii) possible PCOS, histological evidence of PCO with clinical evidence not available (19%) or macroscopic evidence of PCO with clinical evidence of ovarian dysfunction (10%) or clinical diagnosis (9%)	786	56.7 (Mean)	31 years—(range 15–57 years)	United Kingdom	No risk	NA	[61]

*NA* Not available

### Meta-analysis

Available evidence from meta-analyses has reported inconsistent findings with PCOS and breast cancer association [25, 28–30, 40, 58, 59] (Table 2). A modest 9% increase in risk was observed, particularly in European populations, attributed to lifestyle, diet, and healthcare practices [29], while a few other studies found no significant association [25, 28, 30, 40, 58, 59]. It has also been argued that the lack of association observed in several analyses could be due to reduced luteal-phase hormone exposure (estrogen and progesterone) resulting from anovulation [25, 59]. However, it is important to note that there are no mechanistic or animal model-based studies to confirm this argument.

### Cohort studies

A study conducted on 3,566 PCOS women in Taiwan reported a significant trend toward increased breast cancer risk after adjustment for potential confounders, including age, urbanisation, income, and comorbid conditions [31]. However, subsequent analysis using the Monte Carlo random sampling method demonstrated that the mean adjusted hazard ratio (HR) for breast cancer was not statistically different from that of the control group (mean adjusted HR: 1.61, 95% confidence interval (CI): 0.91–2.84), suggesting that the initially observed elevation may have been influenced by random variation in the selection of control subjects. Additionally, elevated AMH/AMH receptor (AMHR2), a hallmark of PCOS, has been positively associated with breast cancer risk and, therefore, considered as a predictive biomarker [56, 57]. Extensive independent cohort studies conducted in Denmark (n = 12,070) [47], Taiwan (n = 8,155) [32], and Sweden (n = 3,493,604) [42] reported that, while women with PCOS have an elevated risk for endometrial cancer, their risk for breast and ovarian cancers is generally comparable to that of the general population.

Variations in findings across meta-analysis and cohort studies may be attributed to differences in case ascertainment, underdiagnosis of PCOS when relying on administrative coding, and inconsistent adjustment for confounders such as body mass index (BMI), parity, and hormonal contraceptive use.

### Evidence from animal and in vitro studies

Estrogen, which is known to be elevated in PCOS, has been shown to promote the proliferation and differentiation of normal breast cells in vitro [62]. Prolonged exposure to estradiol and testosterone implants for 6 months, administered either alone or in combination, has been reported to induce mammary carcinomas in normal rats [63]. Notably, the serum hormonal concentrations observed in this study are comparable to those of experimental PCOS-like animal models [63]. Noroozzadeh et al. [64] reported an approximately 75% higher incidence of mammary tumours in postmenopausal PCOS-like rat models compared to 33.33% in control rats, most likely due to hormonal imbalance during their reproductive life span. However, it is important to note that no compelling evidence for invasive breast cancer formation was observed in this study. Taken together, these findings suggest that ovarian hormones may play a vital role in mammary carcinoma development. Hence, more studies including mechanistic evidence linking prolonged hormonal imbalance and breast cancer development in PCOS animal models are warranted.

#### Comorbidities in PCOS contributing to breast cancer

Evidence from the literature suggests that PCOS is influenced by a range of factors, including hyperandrogenism [24], diabetes [65, 66], hyperinsulinemia [67], insulin resistance [68], obesity [69], higher BMI [70, 71], genetic mutations [72], lifestyle [73], nutrition [74], age [73], exposure to environmental toxicants [75–77], and parity [78], which may collectively or independently contribute to breast cancer risk (Table 3).

**Table 3:** Risk factors associated with breast cancer independent of PCOS

Confounding factor	Risk	Reference
Age	Increase	[73, 79]
Early menarche	Increase	[79, 80]
Late menopause	Increase over 50 years of age	[79, 80]
Delayed pregnancy	Increase over 30 years of age	[79]
Low parity	Increase	[78]
Hormonal	Increase	[81–83]
Genetic mutation		
BRCA1/2	Increase	[84]
FSHR	Increase	[85]
HER2	Increase	[86]
EGFR	Increase	[87]
NME1	Increase	[87]
P53	Increase	[88]
PTEN	Increase	[89]
RB1	Increase	[86, 90]
ATM	Increase	[91]
CDH1	Increase	[65]
PIK3CA	Increase	[86]
Nutrition		
Western dietary pattern	Increase	[74]
Prudent dietary pattern	Decrease	[74]
Obesity	Decrease in premenopausal women Increase in post-menopausal women	[69]
High BMI	Increase risk of ER +/PgR + breast cancer	[70, 71]
Environmental toxicants	Increase	[75, 76]
Lifestyle		
Alcohol consumption	Increase	[73]
Smoking	Increase	[73]
Diabetes	Increase	[65]

#### Hyperandrogenism

Several studies report an increased breast cancer risk in non-PCOS women with free androgen excess [92–94], especially with increased testosterone levels [95]. Women with PCOS are reported to have elevated serum testosterone and free testosterone, and increased androstenedione and DHEA in at least one-third of the subjects [96, 97]. Cumulative data from seven prospective cohort studies analysed by the Endogenous Hormones and Breast Cancer Collaborative Group which included 1699 controls and 767 breast cancer patients, indicated that doubling concentrations of androstenedione (odds ratio (OR): 1.30, 95% CI: 1.10—1.55), DHEA-sulphate (DHEA-S) (OR: 1.17, 95% CI: 1.04—1.32), and testosterone (OR: 1.18, 95% CI: 1.03—1.35) were positively linked with breast cancer risk [98].

A study conducted by Fourkala et al. [99] reported that women had a two-fold higher risk for breast cancer if estrogen receptor α serum bioactivity was increased for more than two years before diagnosis (OR 2.114; 95% CI 1.050–4.425; P = 0.040). Even though AR did not exhibit any significant association with breast cancer, androstenedione (OR, 3.187; 95% CI, 1.738—6.044; P = 0.0003) and testosterone (OR, 2.145; 95% CI, 1.256—3.712; P = 0.006) showed a strong association and almost three-fold increased breast cancer risk, independent of the time of diagnosis. Consistent with these studies, other case–control studies have shown a positive correlation between circulating androgens, hirsutism, and breast cancer incidence [97, 100]. Therefore, the limited inconclusive data and lack of studies on signalling pathways underlying breast oncogenesis in conditions like PCOS require careful investigation.

#### Diabetes, hyperinsulinemia and insulin resistance

Evidence from meta-analysis suggested that elevated glucose [101], hyperinsulinemia [66], and insulin resistance [102], all of which are also associated with type 2 diabetes [66, 103–105], are considered independent risk factors for breast cancer. In addition, both hyperinsulinemia [106] and insulin resistance [107] are reported as significant risk factors for breast cancer [101, 107], independent of general adiposity or body fat distribution [108]. These conditions activate insulin and insulin-like growth factor (IGF) signalling pathways that promote proliferation and suppress apoptosis in mammary tissue [109, 110]. Using a non-obese, muscle IGF-1 receptor-lysine-arginine (MKR) transgenic type 2 diabetes (hyperinsulinemia) mouse model, Novosyadlyy et al. [111] have demonstrated increased mammary cell carcinogenesis via increased phosphorylation and activation of insulin receptor (IR), insulin-like growth factor 1 receptor (IGF-1R), and phosphatidylinositol-triphosphate kinase (PI3K) pathway. Moreover, a study by Bronsveld et al. [112] assessing the correlation between diabetes and clinicopathological breast cancer subtypes indicated that women with diabetes exhibited a greater incidence of progesterone receptor-negative (OR: 2.44, 95% CI: 1.07—5.55), human epidermal growth factor receptor 2 (HER2)-negative (OR: 2.84, 95% CI: 1.11—7.22), and basal-like (OR: 3.14, 95% CI: 1.03—9.60) tumours.

The prevalence of type 2 diabetes is threefold higher in women with PCOS [113, 114], which can be associated with a parallel increase in testosterone levels [115]. Although a fair association of type 2 diabetes with breast cancer risk is observed, limited studies are available that might suggest that PCOS and type 2 diabetes concurrently contribute to increasing breast cancer risk. Cao et al., [116] investigated the combined effect of these conditions on breast cancer and found no association. It is worth mentioning that most studies that investigated the association between PCOS and breast cancer have not factored in the role of glucose levels, hyperinsulinemia, insulin resistance, and type 2 diabetes, making this a significant research gap.

#### Obesity

Obesity, independent of PCOS, is reported to contribute to breast cancer risk [69, 117]. A recent study has shown that BMI was associated with elevated DNA breaks in the breast epithelium in women, as well as in a mouse model [117, 118] suggesting genomic instability in breast tissue that may predispose individuals to breast cancer. Another systematic review and meta-analysis suggested that women who presented with central obesity were 2.4 times more prone to develop breast cancer than those with no history of central obesity (adjusted odds ratio (AOR) = 2.4, 95% CI: 1.35–4.27) [119]. Mechanistically, obesity in PCOS is linked not only to excess estrogen but also to elevated androgen, insulin, and IGF-1 levels [120, 121]. Besides, adipokine dysregulation represents an additional pathway connecting obesity in PCOS to breast cancer. Elevated leptin, frequently observed in obese PCOS women, has been associated with tumour progression and aggressive subtypes, including luminal A and triple-negative breast cancers [122]. In PCOS women, their combined hormonal and metabolic disturbances create a permissive environment for the development and progression of breast cancer [120, 123]. Several cohort studies reinforce this synergistic effect of hormones and growth factors, indicating that women with both PCOS and obesity have greater breast cancer incidence than those with either condition in isolation [31, 47, 60, 124, 125].

#### Genetic predisposition

Both PCOS and breast cancer are complex, polygenic disorders with substantial heritability, suggesting possible overlap in genetic susceptibility. Heritability is estimated up to 70% for PCOS [126] and 35–80% for breast cancer [127], supporting the hypothesis of shared genetic architecture [128]. Genome-wide association studies (GWAS) have identified at least 17 loci associated with PCOS and over 180 variants linked to breast cancer [129]. Mendelian randomisation analyses have further indicated that women with PCOS may have an increased risk of estrogen receptor–positive breast cancer, with single-nucleotide polymorphisms (SNPs) such as rs10739076, rs13164856, and rs11031005 being implicated [130–132]. However, it is important to note that while these variants suggest a possible genetic overlap, direct causal evidence linking PCOS-specific polymorphisms to breast cancer risk remains limited.

Several candidate genes provide mechanistic insight into this potential connection. For instance, cholesterol side-chain cleavage enzyme (CYP11A), which encodes the cholesterol side-chain cleavage enzyme responsible for the first step of steroidogenesis, contains a pentanucleotide repeat polymorphism [(TAAAA)n] associated with PCOS [133, 134]. This same polymorphism has been linked to an increased risk of breast cancer in a Chinese population [135]. Alterations in CYP11A may disrupt normal steroid hormone synthesis, thereby affecting both ovarian physiology and estrogen-driven tumorigenesis. Other genes implicated in both PCOS and breast cancer include hydroxysteroid (17-beta) dehydrogenase 4 (HSD17B4), platelet-derived growth factor receptor alpha (PDGFRA), and high-mobility group box 2 (HMGB2), which play significant roles in steroid metabolism, growth factor signalling, and chromatin remodelling, respectively [136, 137]. Dysregulation in these pathways may contribute to aberrant steroid hormone activity and cellular proliferation. SNPs associated with obesity and metabolic traits, such as insulin and glucose regulation, have been identified as predictors of breast cancer susceptibility [138].

Overexpression of follicle-stimulating hormone receptor (FSHR) in tumour-associated endothelial cells has been described in breast cancer [139], suggesting a link between gonadotropin signalling and tumour angiogenesis. In PCOS women, alterations in key regulatory genes have also been reported. Mutations in the FSHR show strong correlations with elevated breast cancer risk across Asian, African American, and White populations, whereas Erb-B2 receptor tyrosine kinase 2 (ERBB2, also known as HER2) mutations appear more frequent in American Indian or Alaska Native women [140]. Similarly, variants in estrogen receptor 1 (ESR1) and estrogen receptor 2 (ESR2), encoding estrogen receptors α and β, have been reported in PCOS [141–143]. In breast cancer, ESR1 mutations are particularly well described, often leading to ligand-independent receptor activation that promotes estrogen signalling even in the absence of circulating estrogens, a mechanism strongly associated with endocrine therapy resistance and poor prognosis in metastatic disease [144]. Although these mutations have not been directly studied in PCOS-related breast cancer, their presence in both disorders highlights a possible shared pathogenic axis.

Classical breast cancer susceptibility genes also overlap with pathways relevant to PCOS. Germline mutations in breast cancer gene 1 (BRCA1), breast cancer gene 2 (BRCA2), and tumour protein 53 (TP53) disrupt DNA repair and cell cycle regulation, predisposing carriers to breast and ovarian cancers [145–148]. Although such mutations are not typically considered causal in PCOS, shared hormonal environments could exacerbate their tumorigenic potential in affected women. Similarly, activating mutations in HER2/ERBB2, commonly seen in lobular breast cancers, drive aggressive tumour behaviour through enhanced receptor tyrosine kinase signalling [149, 150]. In PCOS, HER2 dysregulation has been less studied, but given the role of growth factor pathways in both disorders, it represents a plausible mechanistic link.

Additional molecular contributors include mutations in the epidermal growth factor receptor (EGFR), which promote uncontrolled cell proliferation [151, 152], and cell division cycle 6 (CDC6), a regulator of DNA replication whose overexpression accelerates tumour growth [153, 154]. Phosphatase and tensin homologue (PTEN) mutations, which impair the PI3K/protein kinase B (AKT) pathway and reduce apoptosis, are also strongly associated with breast carcinogenesis and have been reported in subsets of PCOS patients [145, 155, 156]. Moreover, dysregulation of secreted phosphoprotein 1 (SPP1) and related epigenetic regulators has been shown to promote metastasis and may overlap mechanistically in PCOS and breast cancer [157, 158]. Members of the 17β-hydroxysteroid dehydrogenase (17β-HSD) family further illustrate this connection. 17HSD1 promotes estradiol formation and is associated with poor prognosis in estrogen receptor-positive breast cancer, while hydroxysteroid (17-beta) dehydrogenase 6 (HSD17B6) polymorphisms have been linked to PCOS phenotypes [159, 160].

Matrix metalloproteinases (MMPs) represent another possible molecular link to breast cancer. MMP-9 is a key regulator of extracellular matrix remodelling, facilitating angiogenesis and tumour invasion. High MMP-9 expression has been associated with metastasis and aggressive breast cancer phenotypes [161–163]. Importantly, aberrant MMP-9 expression has also been reported in women with PCOS, suggesting a shared pathogenic mechanism that could predispose this population to cancer development.

#### Menopausal status

Available literature suggests menopausal status as an important parameter to assess the risk of breast cancer [164], especially due to associated factors such as obesity and diabetes [165] in the advanced age group [166]. A strong association between overweight and obesity with breast cancer risk is reported in postmenopausal women, particularly estrogen receptor-positive breast cancer [164, 167, 168]. A prospective cohort study performed in Italy reported that women with a BMI of 30 kg/m^2^ or more had a higher risk of postmenopausal breast cancer (risk ratio (RR): 1.32, 95% CI: 1.00—1.75) [169]. It is noteworthy to mention that postmenopausal women with normal body weight, but a high body fat percentage (normal weight obesity), had a higher risk of breast cancer (HR = 1.19, 95% CI: 1.08–1.31). Wang et al. [170] observed that postmenopausal women with normal BMI had a 15% (95% CI: 10–19%) increased risk of breast cancer for every 5-unit increase in body fat.

Reports on the association between PCOS and breast cancer risk in premenopausal women remain contradictory. Some studies indicate that breast cancer risk is lower in younger women compared to older or postmenopausal women [47, 60], with a potential protective effect attributed to anovulatory cycles [69, 171] and longer menstrual cycles [172]. In contrast, a study involving 1,508 cases and 1,556 controls in Nassau and Suffolk counties, New York, reported a threefold increase in breast cancer risk among premenopausal women with PCOS [27]. Comparatively, in postmenopausal women, elevated BMI is strongly associated with higher circulating estrogen levels due to peripheral aromatisation of androgens in adipose tissue, thereby increasing the risk of estrogen receptor–positive breast cancer [171]. Park et al. [173] reported that postmenopausal women with PCOS and high BMI demonstrated significantly higher susceptibility, a finding attributed to the compounding effects of obesity-induced hormonal imbalance and adipose tissue–derived estrogen production. Meta-analysis conducted by Su et al. [66] suggested a higher risk of breast cancer in patients having diabetes, especially in postmenopausal women. Higher levels of insulin resistance in postmenopausal women are associated with higher breast cancer incidence and all-cause mortality after breast cancer [174]. Collectively, these findings suggest that PCOS may influence breast cancer risk primarily in postmenopausal women, whereas the association in premenopausal populations remains inconsistent.

#### Inflammation

PCOS may predispose women to breast cancer development through multiple interrelated mechanisms involving endocrine, metabolic, immune, and molecular pathways. Elevated levels of C-reactive protein (CRP), interleukin 18 (IL-18), interleukin 6 (IL-6), and tumour necrosis factor-alpha (TNF-α) contribute to systemic inflammation that shapes the tumour microenvironment [175]. Persistent inflammation accelerates adipocyte necrosis, promotes immune cell infiltration, and dysregulates antibody and complement levels, impairing immune surveillance and allowing cancer cells to proliferate and metastasise. Estrogen plays a central role in this context, not only by regulating immune cell polarisation and promoting T helper 17 (Th17) differentiation but also by stimulating IL-6 secretion, thereby amplifying inflammatory responses. TNF-α–induced programmed death ligand 1 (PD-L1) expression within the tumour microenvironment creates an immunosuppressive milieu that inhibits cluster of differentiation 8 (CD8) positive T cell activity, facilitating tumour evasion of immune surveillance [176, 177].

Taken together, these findings suggest that comorbidities implicated in PCOS may play a role in breast cancer risk by influencing steroid hormone metabolism, receptor signalling, DNA repair, and cell proliferation pathways (Table 4). Importantly, each of these factors has been independently associated with cancer development, yet there is limited direct evidence demonstrating that women with PCOS are at higher risk of breast cancer. Thus, they should be regarded as potential shared risk factors, rather than established causal links, highlighting the need for future genetic and molecular studies to clarify these associations.

**Table 4:** Risk factors associated with breast cancer in PCOS women

Risk factors	Risk	Reference
Hirsutism	Increase	[97]
Family history	Increase	[178, 179]
Hormonal imbalance	Increase	[99, 116]
Ovulatory dysfunction	Decrease	[25]
Diabetes	Increase	[113, 114]
Obesity	Increase	[31, 47, 60, 124, 125]
Infertility	Decrease	[180]
Genetic mutation		
MMP9	Increase	[161, 163]
CYP11A	Increase	[135]
ESR1/2	Increase	[141–143]
BRCA1/2	Increase	[145–148]
FSHR	Increase	[140]
EGFR	Increase	[151, 152]
ERBB2	Increase	[140]
TP53	Increase	[145–148]
CDC6	Increase	[153, 154]
PTEN	Increase	[145, 155, 156]
SPP1	Increase	[157, 158]
HSD17B4	Increase	[159, 160]

## PCOS, ovulation induction drugs and assisted reproduction

Among PCOS patients, approximately 70–80% of women experience infertility [181, 182] and often require ovulation induction to achieve pregnancy [183]. The pharmacological interventions commonly includes drugs such as clomiphene and letrozole, or COH with gonadotropin [184]. Ovulation induction protocols encourage the production of a dominant follicle through various mechanisms [185], including preventing the conversion of androgens to estrogens, acting as an antagonist on estrogen receptors, insulin sensitisation, or by direct stimulation of the hypothalamus through gonadotropins [184]. The main complication associated with ovarian stimulation protocols in PCOS women is the altered endocrine profile, which often necessitates the use of higher doses of ovulation induction drugs for prolonged durations to achieve the required ovarian response [184]. Ovulation induction increases serum estradiol levels by approximately two- to three-fold after a single treatment cycle [186, 187] while COH with gonadotropins leads to a transient, but significant increase in serum estradiol levels, similar to those observed in the first trimester of pregnancy [188, 189]. Due to this transitory estrogen surge, there are concerns regarding the possible consequences for short-term or long-term health, specifically for endocrine-sensitive organs like the mammary gland (Table 5). Some of the commonly used ovulation induction drugs and COH employed during ART are discussed below.

**Table 5:** Cancer associated with ovulation induction in infertile women

Cancer	Drug	Risk	References
Ovary	Clomiphene citrate	No risk	[190–193]
	Clomiphene citrate + Human Menopausal Gonadotropin	No risk	[192]
	Gonadotropin	No risk	[191]
	Clomiphene citrate	Increase	[194–198]
	Gonadotropin	Increase	[196]
Breast	Gonadotropin	No risk	[199]
	Gonadotrophin-releasing hormone	No risk	
	Clomiphene citrate	No risk	[192, 199]
	Human Menopausal Gonadotropin	No risk	[192]
	Clomiphene citrate + Human Menopausal Gonadotropin	No risk	
	Clomid	Moderate increase	[200]
	Progesterone	Increase	[199]
	Clomiphene citrate	Increase	[190, 192, 196, 198, 201, 202]
	Gonadotropin	Increase	[196]
	Human Menopausal Gonadotropin	Increase	[203]
	Clomiphene citrate	Decrease	[204]
	Clomiphene citrate + Follicle Stimulating Hormone	Decrease	
Uterus	Clomiphene citrate	Increase	[187, 190, 191, 198]
	Clomiphene citrate	No risk	[205]
Colon	Clomiphene citrate	Moderate risk	[187]
	Gonadotropin	No risk	[187, 206]
	Clomiphene citrate	No risk	[198, 206]
Thyroid	Clomiphene citrate	Moderate risk	[187, 206]
	Gonadotropin	Moderate risk	[207]
	Clomiphene citrate	Increase	[207]
	Progesterone		
	Gonadotropin	No risk	[187, 206]
	Clomiphene citrate	No risk	[198]
Cervix	Clomiphene citrate	Moderate risk	[187]
	Gonadotropin	No risk	
	Clomiphene citrate	No risk	[191, 198]
Melanoma	Clomiphene citrate	Increase	[190, 206]
	Clomiphene citrate	No risk	[198, 207]
	Gonadotropin	Increase	[207]
	Clomiphene citrate	Moderate risk	[187]
	Follicle stimulating hormone	Moderate risk	[208]
	Human Menopausal Gonadotropin	Moderate risk	
	Gonadotropin	No risk	[187, 206]
	Clomiphene citrate	Decrease	[209]
	Human Menopausal Gonadotropin	Decrease	
Non-Hodgkin lymphoma	Ovulation induction	Increase	[190]
	Clomiphene citrate	No risk	

### Metformin

Given the central role of insulin resistance in PCOS pathophysiology, insulin-sensitising drugs have been evaluated to restore ovarian function. Metformin, the most widely used agent, improves menstrual cyclicity, ovulation, and metabolic parameters [210–212]. Mechanistically, metformin improves insulin sensitivity and reduces hyperinsulinemia, thereby lowering ovarian and adrenal androgen secretion, pituitary LH secretion, and increasing hepatic SHBG production [211, 213]. It also exerts direct effects on steroidogenesis and follicular function. Clinical evidence confirms its benefit: a meta-analysis reported higher ovulation (OR: 2.64, 95% CI: 1.85–3.75), clinical pregnancy (OR: 1.98, 95% CI: 1.47–2.65), and live birth rates (OR: 1.59, 95% CI: 1.00–2.51) with metformin versus placebo [214, 215]. Notably, treatment efficacy appears to be BMI-dependent, with obese women demonstrating poorer responses to metformin compared with clomiphene citrate (CC), while non-obese women may experience greater benefit [214].

### Clomiphene citrate

A selective estrogen receptor modulator, has been the standard first-line therapy for anovulatory infertility since the 1960 s [216]. It blocks estrogen receptors at the hypothalamus, increasing gonadotropin-releasing hormone (GnRH) pulsatility and FSH release, thereby stimulating follicle growth. Although CC is effective and inexpensive [217], its anti-estrogenic effects on the endometrium can impair implantation [218]. Due to its long half-life, accumulation with frequent or high-dose use increases the risks of multiple follicular development, multiple pregnancies, and reduced endometrial competence [219]. Therefore, CC is generally prescribed at ≤ 150 mg/day for ≤ 6 cycles [211, 220].

### Letrozole

Letrozole, a third-generation aromatase inhibitor, has emerged as an alternative to CC. By reducing estrogen synthesis, it relieves hypothalamic–pituitary negative feedback, increases FSH secretion, and enhances follicle sensitivity to FSH while supporting endometrial receptivity [218, 221]. Letrozole is usually prescribed at 2.5–7.5 mg/day for 5 days early in the cycle. Clinical trials demonstrate superior efficacy of letrozole over CC by improving ovulation (RR: 1.14), clinical pregnancy (RR: 1.48), and live birth (RR: 1.49) rates [222–224].

### Gonadotropins

Assisted reproduction is considered a third-line option when first-line ovulation induction fails [16]. Women with PCOS are high responders and at increased risk of ovarian hyperstimulation syndrome (OHSS) [225]. Guidelines recommend individualised protocols—starting doses of 100–150 IU gonadotropins, preferably in GnRH antagonist cycles, with final oocyte maturation triggered by GnRH agonists to minimise OHSS risk, followed by elective embryo freezing if needed [226, 227].

## Association of ovulation induction drugs and assisted reproduction with breast cancer risk

Preclinical studies have provided important insights into the potential effects of ovulation-inducing drugs on breast cancer risk. Using a rat model, Richards and Griffith, [228] demonstrated that exposure to CC for 20 days reduced mammary gland cell proliferation. Exposure to CC for more than ten months decreased the probability of developing breast cancer [180], due to the ability of CC to induce apoptosis, as demonstrated in vitro [229] and in vivo [228]. Additionally, CC exhibited direct anti-proliferative action and decreased colony size on estrogen receptor-negative, BRCA1-mutated human breast ductal carcinoma 1937 (HCC1937) cells [230]. Similar results were observed in the estrogen receptor-positive Michigan Cancer Foundation-7 (MCF-7) cell line and the estrogen receptor-negative BT20 cell line [229]. Although there is currently no experimental evidence linking CC to human mammary carcinoma, the secretome of CC-treated human PAX8-positive fallopian tube secretory epithelial cells in vitro was observed to contain a significantly higher abundance of proteins associated with high-grade serous ovarian carcinoma [231]. However, whether CC induces similar response in mammary epithelial cells under hyperandrogenic environment needs to be ascertained.

It is noteworthy to mention that CC was observed to reduce advanced breast cancer in 20 out of 51 patients [196]. Prolonged exposure to CC for more than ten months in patients with ovulatory disorders had decreased breast cancer incidence [180], which is suggested to be due to the structural similarity of CC to tamoxifen [232]. Supporting this, Hecker et al. [233] reported tumour regression in 39% of women with advanced breast cancer treated with CC. Moreover, meta-analyses found no evidence of increased breast cancer risk associated with CC in infertile women [234, 235]. However, high doses of the ovulation induction drug [201], parity [190], number of treatment cycles, and age [236] may increase breast cancer risk in PCOS women. While parous women demonstrated a slightly increased risk, nulliparous women experienced a significantly reduced risk in relation to any fertility treatment (HR: 0.69, 95% CI: 0.49–0.95) as well as specific exposures such as clomiphene use (HR: 0.66, 95% CI: 0.46–0.95) [198, 236]. Brinton et al. [202] found that breast cancer risk was significantly elevated among women who underwent more than 12 cycles of CC and who initiated infertility treatment at 35 years of age or older. In a multicentre case–control study involving 4,575 breast cancer cases aged 35–64 years, Burkman et al. [203] reported a suggestive increase in breast cancer risk among women with a history of infertility, who had used CC for six months (OR: 1.7, 95% CI: 0.9–3.2) or for at least six treatment cycles (OR: 1.7, 95% CI: 0.9–3.0).

Epidemiological evidence regarding the association between metformin use and breast cancer risk or mortality has been inconsistent. Several studies have reported a reduced risk of breast cancer incidence and/or cancer-related mortality among individuals with diabetes treated with metformin compared to those receiving other antidiabetic agents [237–239]. Furthermore, preclinical studies demonstrate anti-proliferative effects in breast cancer, largely attributed to its role as an adenosine monophosphate-activated protein kinase (AMPK) activator and thereby inhibiting mechanistic target of rapamycin (mTOR) [240–242]. In contrast, other investigations have not observed any significant impact of metformin on breast cancer risk [243–245]. No reports are currently available in the literature regarding the association between metformin use and breast cancer risk or its effects on mammary cells in women with PCOS. However, earlier studies have observed adverse effects on other cell types [246–248], especially germ cells [249–251]. Considering the AMH and testosterone-lowering impact of metformin, elucidating its role in mammary gland function in PCOS women represents an important area for future research.

Existing studies in the literature offer inconsistent reports regarding the relationship between COH and the risk of breast cancer. Few studies suggest a potential association between infertility treatments and increased risk of cancer [192, 198, 202], while others report no significant association [236, 252–255]. Similarly, a modest increase in the incidence of breast cancer was observed in patients who received fertility medication for in vitro fertilization (IVF) [256]. On the contrary, Terry et al. [180] reported that women with ovulatory disorder-related infertility who underwent ovulation-induction had the lowest incidence of breast cancer (covariate-adjusted HR: 0.60; 95% CI: 0.42—0.85). A large population-based cohort study found no increased breast cancer risk associated with infertility treatments after up to 5 years of follow-up, relative to age-specific general population rates [252]. Liat et al. [192], reported that gonadotropin exposure was not associated with increased breast cancer risk in a cohort of Israeli women with over 30 years of follow-up. Similar findings were observed in a study of 1,340,211 Swedish women undergoing assisted reproduction treatment, where breast cancer incidence was comparable to that of the general population. Additionally, no malignancy risk was found in association with the IVF procedure or GnRH analogue use, whereas a decreased risk was reported following progestogen use [236]. A study conducted by Jensen et al., [199] reported that there is no overall increase in breast cancer risk in 54,362 infertile Danish women treated with gonadotropins, human chorionic gonadotropin (hCG), or GnRH analogues. However, progesterone use among these patients was associated with a higher risk (RR, 3.36; 95% CI, 1.3–8.6). All progesterone-exposed cases had also received multiple fertility drugs, making it difficult to attribute the increased risk specifically to progesterone.

Intuitively, prolonged exposure to drugs that elevate serum estradiol levels at advanced maternal age may increase breast cancer risk [253, 257, 258]. Vassard et al. [258] highlighted a strong positive correlation between age at initiation of infertility treatment, particularly in women over 40 years, and breast cancer. Similarly, a cohort study of 3,375 women demonstrated a possible association between IVF treatment and breast cancer**,** with a higher risk observed among women aged ≥ 40 years at treatment and those undergoing ≥ 4 IVF cycles [257]. In contrast, Van den Belt-Dusebout et al. [253] did not find any correlation between the number of IVF cycles and breast cancer, although an increase in age-related risk was observed for women older than 35 years. While no overall increase in the risk of breast cancer was linked to IVF medications, a study involving over 250,000 women reported that the number of treatment cycles increased the frequency of in situ breast carcinoma [259]. Conversely, no association was found between the number of treatment cycles and breast cancer risk in a retrospective, population-based cohort study [255].

Interpretation of these findings is limited by several methodological challenges. Many studies are observational and non-randomised, with potential for detection bias (due to increased clinical surveillance of infertility patients), confounding factors (such as parity, contraceptive use, reproductive history, and socioeconomic factors), and heterogeneity in study design. Moreover, overlapping drug regimens make it difficult to isolate the effect of individual agents on the breast cancer risk (e.g., progesterone vs gonadotropins). Some studies had relatively short follow-up periods, while others reported increased risk only decades after exposure, underscoring the need for long-term surveillance, as these effects may manifest after prolonged intervals.

## Conclusions and limitations

From the available literature, the association between PCOS and the risk of breast cancer appears to be complex and multifaceted, and the specific confounder influencing this relationship remains unclear. While the hormonal imbalances associated with PCOS, including hyperandrogenism, insulin resistance, and disrupted estrogen-progesterone dynamics, may contribute to an altered breast development process and potentially affect breast cancer risk, the evidence remains inconsistent. Additionally, fertility drugs and COH transiently alter the endocrine profile, which may influence breast cancer risk, though studies have reported conflicting results. Interpretation of the existing evidence is limited by several factors, including inadequate stratification of the underlying condition, small sample size, heterogeneity in treatment protocol (type of drug used, dosage, and number of cycles), and lack of consideration of lifestyle and metabolic factors such as diet and obesity. These limitations make it difficult to draw definitive conclusions regarding the magnitude of breast cancer risk associated with PCOS and fertility treatments. Given the complex interplay between these factors, further research is crucial to elucidate the underlying mechanisms and clarify the long-term health implications for women with PCOS. As the use of assisted reproductive technology continues to increase, it is essential to balance the benefits of ovulation induction with careful evaluation of potential risks, ensuring that women with PCOS receive informed care customised to their individual health needs.

### Scientific gaps and potential research directions

PCOS is a complex condition with heterogenous phenotypic, biochemical and ovarian function manifestations. Several biochemical parameters such as hyperinsulinemia and type 2 diabetes, both of which are frequently associated with PCOS can be predisposing factors to breast cancer independently. However, the extent to which these comorbidities contribute to breast cancer risk in PCOS women is understudied. Another important aspect which needs to be addressed is the potential long-term effects of drugs such as letrozole and metformin, which are commonly prescribed for ovulation induction and management of PCOS. Their possible influence on the breast cancer risk needs to be assessed. Systematic multicentric cohort studies with extended follow-up are required for better understanding of the potential association between infertility treatments and breast cancer in women with PCOS. Establishing national infertility treatment registries that document treatment protocol, type of drug used, dose and duration of treatment, and subsequent health outcomes would greatly facilitate assessment of cancer risk, especially in the PCOS population. Mechanistic understanding of breast cancer development in the context of PCOS still remains poorly understood. Even though true PCOS experimental models are yet to be established, future studies using PCOS mouse models to evaluate whether hyperandrogenism contributes to spontaneous breast tumorigenesis could provide more physiologically relevant insights into cancer initiation.

## Data Availability

Not applicable.

## Abbreviations

PCOS: Polycystic ovary syndrome
WHO: World Health Organization
COH: Controlled ovarian hyperstimulation.
LH: Luteinizing hormone
DALY: Disability-adjusted life years
GLOBOCAN: Global Cancer Observatory
DENND1A: Denn domain-containing protein 1A
BMI: Body mass index
EGF: Epidermal growth factor
IGF: Insulin-like growth factor
IGF-1R: Insulin-like growth factor 1 receptor
SHBG: Sex hormone binding globulin
AMH: Anti-Müllerian hormone
DHEA: Dehydroepiandrosterone
DHEA-S: DHEA-sulphate
AR: Androgen receptor
MKR: Muscle IGF-1 receptor-lysine-arginine
IR: Insulin receptor
PI3K: Phosphatidylinositol 3-kinase
HER2: Human epidermal growth factor receptor 2
SNP: Single-nucleotide polymorphisms
CYP11A: Cholesterol side-chain cleavage enzyme
FSH: Follicle-stimulating hormone
FSHR: Follicle-stimulating hormone receptor
ERBB2: Erb-B2 receptor tyrosine kinase 2
HSD17B4/6: Hydroxysteroid (17-beta) dehydrogenase 4/6
PDGFRA: Platelet-derived growth factor receptor alpha
HMGB2: High-mobility group box 2
TP53: Tumour protein 53
ESR1/2: Estrogen receptor 1/2
MMP: Matrix metalloproteinases
AMPK: Adenosine monophosphate-activated protein kinase
mTOR: Mechanistic target of rapamycin
CC: Clomiphene citrate
IVF: *In-vitro* Fertilisation
CI: Confidence interval
OR: Odds ratio
RR: Risk ratio
AOR: Adjusted odds ratio
GWAS: Genome-wide association studies
HR: Hazard ratio
GnRH: Gonadotropin-releasing hormone
BRCA1/2: Breast cancer gene 1/2
EGFR: Epidermal growth factor receptor
CDC6: Cell division cycle 6
PTEN: Phosphatase and tensin homologue
SPP1: Secreted phosphoprotein 1
AKT: Protein kinase B
17HSD: 17β-Hydroxysteroid dehydrogenase
PD-L1: Programmed death ligand 1
HCC1937: Human breast ductal carcinoma 1937
MCF-7: Michigan Cancer Foundation-7
hCG: Human chorionic gonadotropin
CRP: C-reactive protein
IL-6/18: Interleukin – 6/18
Th17: T helper 17
CD8: Cluster of Differentiation 8
TNF-α: Tumour necrosis factor-alpha
NIH: National Institute of Health
ESHRE: European Society of Human Reproduction and Embryology
ASRM: American Society for Reproductive Medicine
OHSS: Ovarian hyperstimulation syndrome
